# Effects of Phytogenic Feed Additive on Production Performance, Slaughtering Performance, Meat Quality, and Intestinal Flora of White-Feathered Broilers

**DOI:** 10.3390/vetsci12050396

**Published:** 2025-04-22

**Authors:** Jianming Ren, Siyu Ren, Haochi Yang, Peng Ji

**Affiliations:** 1 College of Chemistry and Life Sciences, Gansu Normal University for Nationalities, No. 233, Zhihema Road, Hezuo 747000, China; 2 Department of Traditional Chinese Veterinary Medicine, College of Veterinary Medicine, Gansu Agricultural University, No. 1 Yingmen Village, Anning District, Lanzhou 730070, China; 15693872517@163.com (S.R.); 1073323140026@st.gsau.edu.cn (H.Y.)

**Keywords:** average daily gain, meat quality, 16S rDNA, beneficial flora, correlation analysis

## Abstract

This study systematically evaluates the effects of dietary supplementation with phytogenic feed additive (0.2%, 0.4%, and 0.8%) on white-feathered broilers (n = 88) through a 42-day controlled trial with the weight of approximately 50 g. The experimental design incorporates a triplicate-group-replicated protocol with daily feed intake monitoring, culminating in comprehensive assessments of the growth performance, slaughter traits, meat quality, and cecal microbiome dynamics. The results demonstrated that the 0.8% supplementation significantly enhanced average daily weight gain (*p* < 0.05), optimized meat characteristics (elevated the redness of meat, reduced pH; *p* < 0.05), and restructured cecal microbiota by enriching *Deinococcus-Thermus, Bacteroidetes, Actinobacteria,* and *Cyanobacteria* (*p* < 0.05). Based on microbiota-based functional prediction analyses (COG/KEGG/MetaCyc), phytogenic feed additive significantly activated lipid metabolism pathways in broilers. The immunomodulatory correlations between *Deinococcus/Thermus/Cyanobacteria* and immune indicators suggested their potential immune-enhancing effects mediated through host immune regulation. The findings established the 0.8% phytogenic feed additive as a multifunctional phytogenic additive that synchronously improves zootechnical performance, meat quality, and microbiome homeostasis, offering a scientifically validated strategy for antibiotic-free precision nutrition in sustainable poultry production.

## Simple Summary

This study evaluated the effects of phytogenic feed additive on the white-feathered broilers. Eighty-eight broilers were divided into four groups and fed for forty-two days. The results showed that the 0.8% phytogenic feed additive optimized the broiler health, growth, and meat quality, supporting the use in poultry farming, and it particularly increased the beneficial bacteria, like *Deinococcus-Thermus* and *Cyanobacteria*, which were linked to enhanced immunity and lipid metabolism.

## 1. Introduction

As an important component of China’s agricultural production, the livestock industry’s economic output constitutes approximately one-third of the total agricultural output, thereby playing a pivotal role in bolstering the national economy and ensuring the welfare of the population. The broiler industry, a principal segment of the livestock sector, has a research market share of approximately 20%. White-feathered broilers have emerged as a predominant breed in the poultry industry owing to their swift growth and affordability. However, these broilers exhibit diminished capacity to resist diseases and are prone to infections by pathogenic microorganisms, and thus antibiotics are widely utilized in their husbandry [1]. Given the escalating issues of antibiotic resistance and residue accumulation, the government has promulgated regulations aimed at “eliminating antibiotics from feed and restricting their use in therapy”. Consequently, the investigation, development, and implementation of viable alternatives to antibiotics have become imperative.

In recent years, the incorporation of traditional Chinese medicine (TCM) in the husbandry of livestock and poultry has witnessed a steady increment owing to the natural properties of TCM and its reduced propensity to induce drug resistance. This development offers innovative perspectives for the pursuit of sustainable and health-oriented broiler production practices. TCM ingredients are derived from a diverse array of botanical sources and are known for their rich content of bioactive compounds, including flavonoids [2], polysaccharides [3], and alkaloids [4]. Preliminary research has indicated that phytogenic feed additives mainly include *Radix Codonopsis pilosulae*, *Radix Astragali*, *Poria*, *Massa Medicata Fermentata*, *Rhizoma Atractylodis macrocephalae*, *Crataegus pinnatifida*, *Fructus Hordei germinatus Preparata*, and *Alpinia katsumadai*. The active principles of phytogenic feed additives include total polysaccharides and total flavonoids, and they ameliorate the growth performance in western crossbred cattle, enhance digestive and absorptive capabilities, and bolster antioxidant defenses [5]. Similarly, the beneficial effects of *Astragalus* [6] and *Codonopsis* polysaccharides [7] are integrated into the breeding regimens of chickens and ducks. *Codonopsis* pilosula, recognized as the dried root of *Platycodon* grandiflorum, is revered for its ability to strengthen the spleen, benefit the lungs, nourish the blood, and generate body fluids. Its chemical composition is intricate, encompassing polysaccharides, flavonoids, saponins, and other active constituents [8], which significantly enhance an organism’s antioxidant capacity and thereby improve overall health [9]. *Astragalus*, as a cornerstone of TCM, is clinically employed to augment the immune response and shield poultry from infections by pathogenic microorganisms [10]. Fu et al. demonstrated that *Atractylodes macrocephala* polysaccharides (AMP)—a principal bioactive constituent of this traditional Chinese medicinal herb is *Rhizoma Atractylodis macrocephalae*—significantly enhance growth performance and modulate immune responses in animal models [11]. Emerging research indicates that dietary supplementation with *Poria cocos* polysaccharides (PCP) significantly improves the growth performance of broilers by preserving intestinal mucosal integrity, thereby elucidating a potential mechanism for its growth-promoting effects in poultry production [12]. While previous studies predominantly focus on the clinical application of monomeric compounds from TCM as feed additives, their industrial implementation remains constrained by complex extraction protocols and prohibitive production costs. Notably, the source herbs of these investigated monomers align pharmacologically with the composition of Qiangzhuang Powder, a canonical veterinary formula documented in the Chinese Veterinary Pharmacopoeia (2020 edition). To address these technological and economic barriers, we developed a novel modified formula, phytogenic feed additive, through systematic optimization of the original Qiangzhuang Powder formulation.

The intestinal microbiota is instrumental in sustaining the proliferation of intestinal epithelial cells, mounting a defense against pathogenic organisms, synthesizing secondary metabolites, and facilitating the digestion of complex carbohydrates [13]. Dysbiosis within the intestinal microbiome can promote compromised nutrient assimilation, diminish immune function, and impair intestinal barrier integrity, potentially intensifying the severity of disease manifestations [14]. Herbal medicines exert a bidirectional interaction with the intestinal microbiota, modulating the composition and functionality of the intestinal microbiota while concurrently being subject to microbial metabolism. Microbial metabolites resulting from these interactions can exert substantial pharmacological effects on host organisms, particularly with respect to intestinal barrier function [15]. Consequently, the modulation of intestinal microbiota equilibrium emerges as a paramount strategy for enhancing the health status of broilers.

The effects of the traditional Chinese medicine compound phytogenic feed additive on the growth performance, carcass characteristics, meat quality, and intestinal microbiota in the white-feathered broilers are assessed in this study. A comparative analysis was conducted across different dosages of phytogenic feed additive and a control group to validate its prophylactic potential in enhancing the weight gain and meat quality of the broiler.

## 2. Materials and Methods

### 2.1. Ethical Statement

In this study, the animal welfare and experimental procedures were approved according to the guidelines for animal care and use and by the Animal Ethics Committee of Gansu Agricultural University (GSAU-Eth-VMC-2021-360).

### 2.2. Test Materials

Phytogenic feed additives, mainly including *Radix Codonopsis pilosulae* (200 g), *Radix Astragali* (200 g), *Poria* (150 g), *Rhizoma Atractylodis macrocephalae* (100 g), *Fructus Hordei germinatus Preparata* (70 g), *Massa Medicata Fermentata* (70 g), *Crataegus pinnatifida* (70 g), and *Alpinia katsumadai* (140 g), were mixed to formulate. The broilers were fed with the basal diet (Charoen Pokphand Group (Lanzhou) Co., Ltd., China Zhengda 510 small chicken feed and Zhengda 511 medium chicken feed [16]). Each plant in the formula was thoroughly crushed, passed through a 100-mesh plug, thoroughly mixed, and then mixed with the base diet in different proportions (0.2%, 0.4%, and 0.8%).

The main raw materials of Zhengda 510 and 511 feed are corn, soybean oil, soybean meal, rapeseed meal (cake), cottonseed meal (cake), corn protein powder, stone powder, sodium chloride, vitamins, mineral elements, and so on.

China Zhengda 510 (1–21 d): moisture ≤ 14.00, CP ≥ 20.00, Ca: 0.60–1.20, TP ≥ 0.50, NaCl: 0.20–0.80, CF ≤ 6.00, ash ≤ 8.00, and eggs + cystine ≥ 0.74.

China Zhengda 511 (22–42 d): moisture ≤ 14.00, CP ≥ 19.00, Ca: 0.60–1.20, TP ≥ 0.40, NaCl: 0.20–0.80, CF ≤ 6.00, ash ≤ 8.00, and eggs + cystine ≥ 0.71.

### 2.3. Experimental Design and Feeding Management

A total of 88 healthy 1-day-old white-feathered broilers (44 males and 44 females) were procured from the Shandong Dacheng Poultry Farming Group in China and randomly allocated to 4 experimental groups. The groups consisted of the control group (C group), which was fed with a basal diet without any supplementation, and three treatment groups, which received a basal diet supplemented with phytogenic feed additive at concentrations of 0.2% (low-dose group, L), 0.4% (medium-dose group, M), and 0.8% (high-dose group, H). The control group contained two replicates, whereas the treatment groups had three replicates (each replicate included 5–8 chickens). The experimental duration was 42 days. White-feathered broilers of 1~21 days old were fed Zhengda 510 small chicken feed, and white-feathered broilers of 22~42 days old were fed Zhengda 511 medium chicken feed. Under uniform housing conditions, broilers in each group had ad libitum access to water through nipple drinkers, were manually fed, and had their feces removed daily. The housing environment was managed with proper ventilation and air exchange. For 1~3-day-old white-feathered broilers, temperature was controlled at 33~35 °C, and humidity at about 70%. For 4~20-day-old white-feathered broilers, temperature was controlled at about 23 °C, and humidity was maintained at about 60~65%. For white-feathered broilers aged 21~35 days, the temperature was maintained at about 23 °C, and the air humidity was maintained at 55~60%. Regarding white-feathered broilers after 35 days of age, the humidity was maintained at 50~55%, and the temperature was about 23 °C. The broilers were subjected to routine vaccination protocols based on the standard procedures.

### 2.4. Measurement of the Growth Performance

At the start of the experiment, the initial body weight of each one-day-old broiler was ascertained and documented. Subsequently, the final body weight was determined after a 42-day experimental period, and the broilers were subjected to 12 h of food and water fasting before weighing. Throughout the duration of the study, the feed consumption and fecal output of the broilers in each replicate were monitored daily and documented, and their weight was precisely measured. The average daily feed intake (ADFI), the average daily weight gain (ADG), and the feed-to-body-weight-gain ratio (FCR) were derived from the daily records and calculated for each replicate (NY/T 828-2004). These parameters are critical for assessing the growth performance and the feed efficiency of the broilers under experimental conditions:

(1) $$ADFI(g/day)=\frac{Feed\;Provided\;\left(g\right)-Residual\;Feed\left(g\right)}{Number\;of\;Animals\times Days}$$

(2) $$ADG=\frac{FBW-IBW}{d}$$

(3) $$FCR=\frac{Total\;Feed\;Intake\;(kg)}{Body\;Weight\;Gain\;(kg)}$$

### 2.5. Sample Collection

At the end of the experimental period, white-feathered broilers were euthanized with sodium pentobarbital. Eleven white-feathered broilers per group were randomly selected, and some samples, including breast muscle, leg muscle, abdominal fat, and cecal contents (sterile collection), were collected from the selected broilers for further analytical evaluation.

### 2.6. Measurement of the Carcass Performance

The breast muscle, leg muscle, and abdominal fat were separated and weighed for recording, and the carcass percentage, half carcass percentage, full carcass percentage, breast muscle percentage, leg muscle percentage, and abdominal fat percentage were calculated. The calculation of the carcass performance in poultry production performance was in accordance with NY/T823-2022 Nomenclature and Metric Calculation Methods.

### 2.7. Determination of the Meat Quality

One side of the pectoral muscle was trimmed according to the method of NY/T1333-2007 Livestock and Poultry Meat Quality Determination. The pH measurement device was used to determine the pH at 3 different positions of the pectoral muscle, and the color meter was used to determine the L*, a*, and b* of the pectoral muscle. An appropriate amount of pectoral muscle was wrapped in gauze and pressed at 35 kg using 8 layers of filter paper on a YYW-2 strain-controlled non-lateral pressure gauge for 5 min. After removal, it was weighed to calculate the water loss rate under pressure. Another portion was placed in an aluminum foil bag, heated in a water bath (75~80 °C) until the core temperature reached 70 °C, then cooled to room temperature and weighed to determine the cooking loss rate.

### 2.8. Measurement of Cecal Contents

The cecal contents of 10 white-feathered broilers from both the control group and the optimal dose group were collected for intestinal microbiota analysis. The specific methods are described as follows: The PCR reaction system was configured with 30 ng of acceptable quality genomic DNA samples and appropriate fusion primers. The PCR amplification was performed by setting the PCR reaction parameters. The PCR amplification products were purified using Agencourt AMPure XP magnetic beads, dissolved in the elution buffer, labeled, and the library construction was completed. The fragment range and concentration of the libraries were determined using an Agilent 2100 Bioanalyzer. The qualified libraries were sequenced on the sequencer according to the insert size. The data were filtered, and the remaining clean data were used for later analysis. The reads were spliced into tags by the overlap relationship between the reads, the tags were clustered into OTUs and compared with the database, and the species were annotated. Based on the results of the OTUs and the annotations, we performed the complexity analysis on the samples, the analysis of the species differences between the groups, and the analysis of the associations and the prediction of the model, and so on (the research method was detected and provided by BGI China).

### 2.9. Statistics and Data Analysis

One-way ANOVA was performed using Excel 2016 and GraphPad Prism 8.0.2 software, and Tukey’s method was used for multiple comparisons of the means of each group. The results were expressed as mean ± standard deviation, and *p* < 0.05 indicated significant differences, while *p* > 0.05 indicated nonsignificant differences.

## 3. Results

### 3.1. Impact of Phytogenic Feed Additive on the Growth Performance of White-Feathered Broilers

As depicted in Table 1, the average daily weight gain of white-feathered broilers in the L, M, and H groups was significantly higher than that of the C group (*p* < 0.05). In addition, compared with the C group, the material-to-weight ratio of white-feathered broilers in the L, M, and H groups decreased nonsignificantly (*p* > 0.05). The findings imply that the average daily weight gain of white-feathered broilers was positively influenced by the inclusion of different concentrations of phytogenic feed additive in their basal diets, with the most beneficial effects being received by the H group.

### 3.2. Impact of Phytogenic Feed Additive on the Slaughtering Performance of White-Feathered Broilers

As presented in Table 2, the dressing performance of white-feathered broilers in the H group surpassed than that of the C group, suggesting that dietary supplementation with the 0.8% phytogenic feed additive improved the slaughtering efficiency of the broilers.

### 3.3. Impact of Phytogenic Feed Additive on the Quality of Meat from White-Feathered Broilers

As presented in Table 3, the a* value of white-feathered broilers in the H group was significantly greater (*p* < 0.05) and the pH value was significantly lower (*p* < 0.05) than those of the C group at 1 and 24 h post-slaughter intervals. Meanwhile, the L* and b* values and pressurized water loss rate showed a nonsignificant downward trend compared with the C group (*p* > 0.05). These results indicated that the inclusion of the 0.8% phytogenic feed additive in the diet could enhance the meat quality attributes of white-feathered broilers.

### 3.4. Impact of Phytogenic Feed Additive on the Intestinal Flora of White-Feathered Broilers

#### 3.4.1. Alpha-Diversity of the Intestinal Flora of White-Feathered Broilers

**Figure 1:**
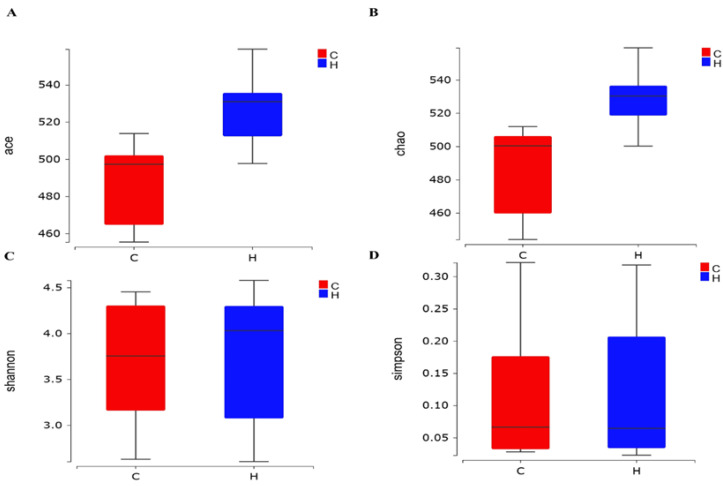
Effect of phytogenic feed additive on the alpha-diversity of the intestinal microbiota in white-feathered broilers. (**A**) Ace index, (**B**) Chao index, (**C**) Shannon index, and (**D**) Simpson index.

The α-diversity analysis of two groups of samples can accurately reflect the species and structural diversity of the microbial community in the rumen. The rumen microbial Chao and ace indices of the H group significantly differ from those of the C group (*p* < 0.05), indicating that the addition of phytogenic feed additive had a marked effect on the relative abundance of the intestinal microbial flora of the broilers (Figure 1A–D).

#### 3.4.2. β-Diversity of Intestinal Flora in White-Feathered Broilers

**Figure 2:**
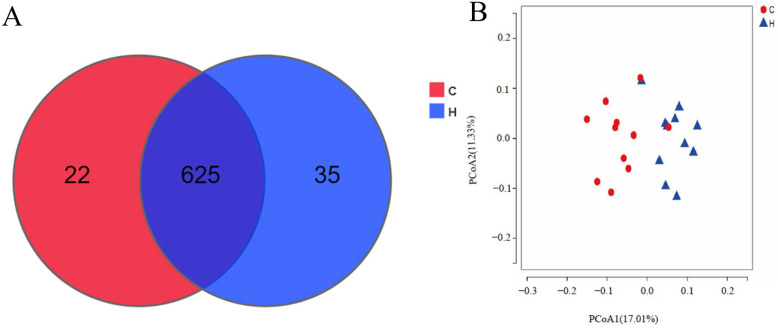
Effect of phytogenic feed additive on the β-diversity of the intestinal microbiota in white-feathered broilers. (**A**) OTUs clustering diagram and (**B**) PCoA clustering diagram.

As illustrated in Figure 2A, the total number of operational taxonomic units (OTUs) in group C was 647, and the total number of OTUs in the H group was 660. Principal coordinates analysis demonstrated that the flora between the H and C groups were distinctly separated but showed a notable degree of similarity (Figure 2). The results show that the phytogenic feed additive exerted a considerable influence on the regulation of the intestinal flora of the broilers.

### 3.5. Composition and Structure of the Intestinal Flora at the Phylum and Genus Levels in White-Feathered Broilers

**Figure 3:**
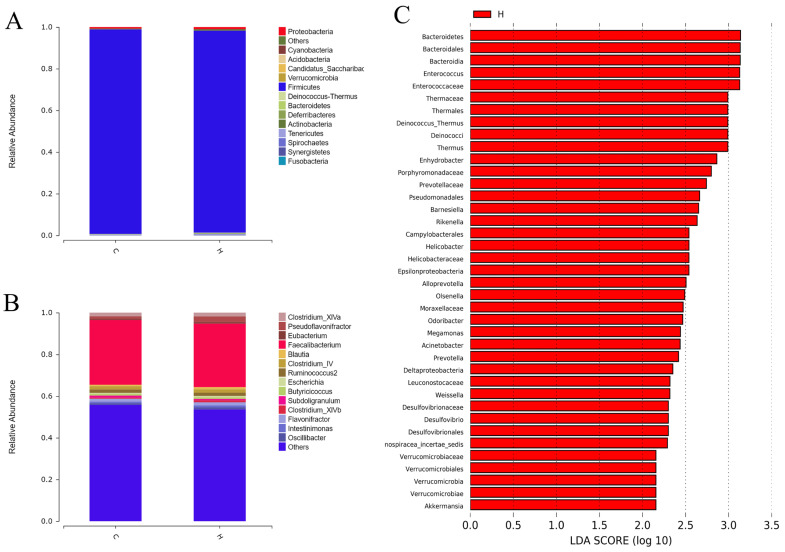
Effects of phytogenic feed additive on the phylum- and genus-level composition and the intestinal microbiota structure in white-feathered broilers. (**A**) Phylum diagram, (**B**) genus diagram, and (**C**) LEfSe diagram.

Furthermore, no notable discrepancies were identified between the two groups with respect to the core microbiota at the phylum and genus levels (Figure 3A–C; *p* > 0.05). However, the relative abundance of four phyla, namely, *Deinococcus-Thermus*, *Bacteroidetes*, *Actinobacteria*, and *Cyanobacteria*, was significantly higher at the phylum level in the H group (Figure 3A,C; *p* < 0.05). This result suggests that the phytogenic feed additive might increase the relative abundance of beneficial bacteria in white-feathered broilers.

### 3.6. Predictive Analysis of the COG, KEGG, and Metacyc Pathway Results

**Figure 4:**
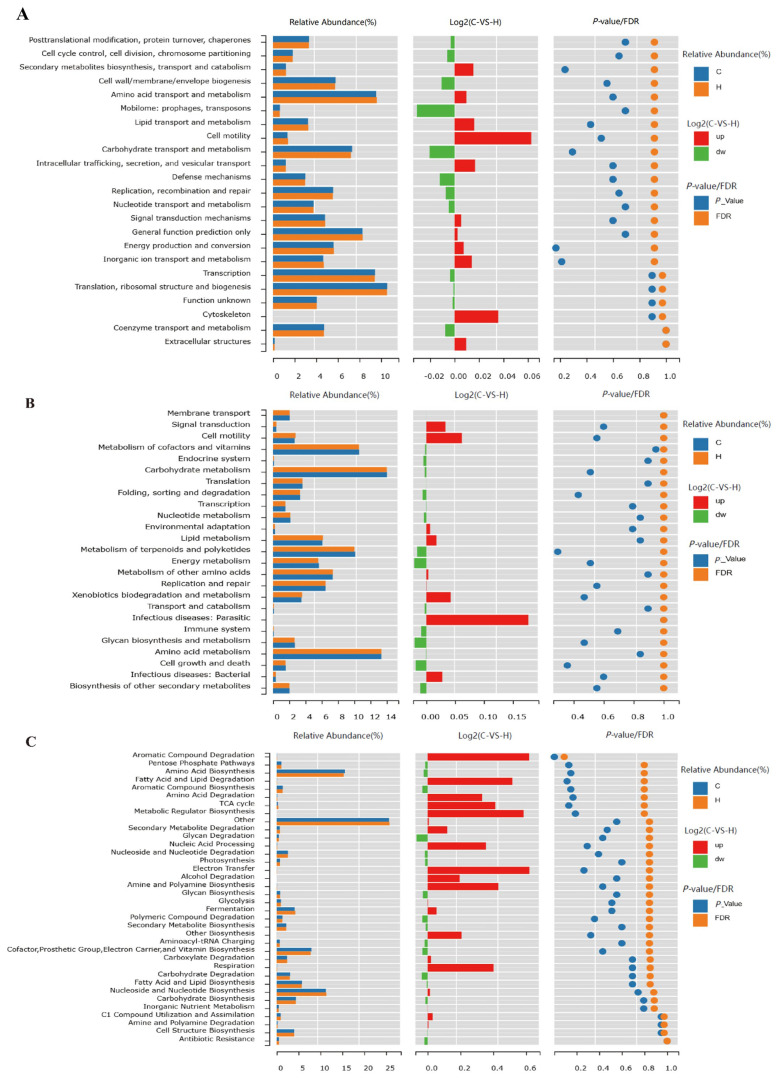
Prediction of the effects of phytogenic feed additive on COG, KEGG, and Metacyc pathways. (**A**) COG access road forecast, (**B**) KEGG access road forecast, and (**C**) Metacyc access road forecast.

Predictive analysis of the COG, KEGG, and Metacyc pathway results in the intestinal contents of white-feathered broiler chickens by 16S high-throughput sequencing revealed that phytogenic feed additive had an upregulatory effect on the pathways of lipid transport and metabolism, lipid metabolism, and fatty acid and lipid degradation in the intestinal flora of white-feathered broiler chickens, respectively (Figure 4A–C).

### 3.7. Analysis of the Correlation Between Clinically Relevant Indices and Gut Contents in White-Feathered Broilers

**Figure 5:**
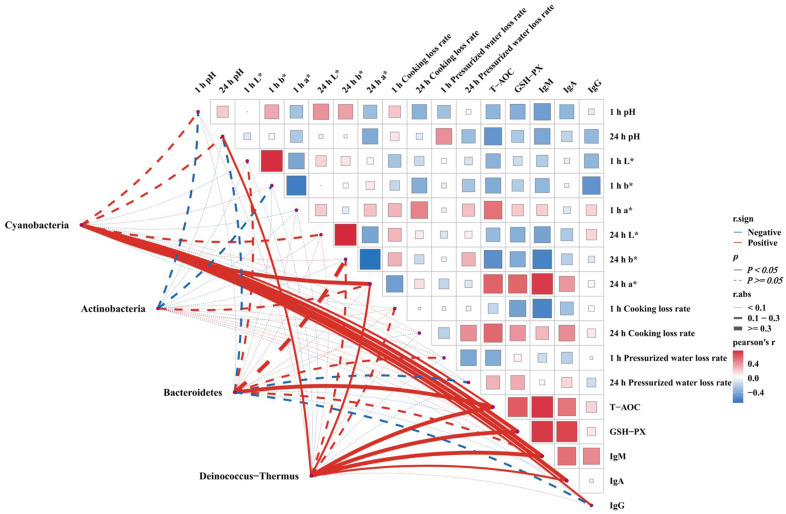
Correlation analysis between clinical indicators and significant differences in cecal microbiota in white-feathered broiler chickens. L* represents the brightness value of the chicken breast, b* represents the yellowness value of the chicken breast, and a* represents the redness value of the chicken breast. The red indicates a positive correlation and blue indicates a negative correlation.

The Spearman’s correlation analysis results of the antioxidant-, immune- [16], and meat-quality-related indicators with gut differential phyla showed that the two differential phyla, *Cyanobacteria* and *Deinococcus-Thermus*, were strongly correlated with the immune indicators of IgA and IgM, respectively (r > 0.5, *p* < 0.05), suggesting that the relative abundance of these two phyla changes indirectly regulated the immunity level of white-feathered broilers. The results further indicated that the additive may not only directly regulate the gut flora of white-feathered broilers, but also indirectly regulate the immunity of white-feathered broilers through the relative abundance of the gut flora of white-feathered broilers (Figure 5).

## 4. Discussion

The practice of TCM allows for the regulation of all aspects of the body of livestock and poultry through the synergistic effects of various bioactive ingredients in TCM [17]. These effects improve animal growth performance and meat quality. The findings of the present study indicated that the incorporation of varying concentrations of phytogenic feed additive into broiler basal diets increased the average daily feed intake and average daily weight gain of broilers while reducing the feed-to-weight ratio. To date, the research exploring the potential applications of phytogenic feed additive in livestock and poultry farming is limited. Nevertheless, a study demonstrated that the average daily weight gain of six-month-old western hybrid cattle was considerably enhanced by the spleen-healthy and anti-conductivity Chinese medicine compound Ren Jianming [5]. Additionally, the addition of Poria cocos polysaccharide to the diet exerted a beneficial effect on the growth performance, immunity, and cecum microbiota of piglets [18]. The present study demonstrated that the dietary addition of Astragalus considerably enhanced the performance and antioxidant capacities of broilers [19]. The addition of ginseng stem and leaf extract to the diet exerted beneficial effects on a number of parameters, including body weight, meat quality, antioxidant capacity, immunity, and blood lipids, in broilers [20]. The addition of fermented ginseng lingbaijusan to the diet can considerably enhance the growth performance of yellow-feathered broilers [21]. This beneficial effect of phytogenic feed additives may be attributed to multiple Chinese medicinal ingredients, including polysaccharides, flavonoids, and saponins, which may fortify the stomach and facilitate digestion, and thereby promote growth.

In addition to growth performance, slaughter performance is an important indicator affecting the breeding of white-feathered broiler chickens. The slaughter rate, semi-clearance rate, and full-clearance rate are key indices of chicken meat production. The slaughter performance of broilers in poultry farming should be enhanced. The results of the present study demonstrated that the addition of the 0.8% phytogenic feed additive to the feed increased the slaughtering, semi-clearance, full-clearance, leg muscle, and breast muscle rates of white-feathered broilers. The observed enhancement in slaughter performance parameters aligns with previous findings in other poultry species. Notably, Another study showed that adding traditional Chinese medicine to the diet can significantly improve the growth and slaughter performance of Nandu Yellow chickens [22]. The incorporation of mulberry leaves into the diet of poultry enhanced the growth performance and slaughtering rate [23]. Astragalus polysaccharides given to broilers resulted in a significant increase in leg muscle percentage and abdominal fat percentage [24].

The production of broilers has become an essential commercial undertaking, and efforts have been devoted to enhancing the quality of the meat produced by modifying various characteristics of the meat [25]. The most important and perceptible characteristics of meat are its appearance, texture, juiciness, moisture, hardness, tenderness, odor, and flavor, which influence consumers’ initial and final quality assessments before and after they purchase meat products. Among the numerous parameters that define meat quality, consumers’ overall perception of meat quality is directly reflected by meat color [26]. Consequently, the primary objective of improving meat quality is to enhance meat color. The principal test for improving the physicochemical indicators of meat is testing the muscle tethering power, which is directly influenced by the rate of water loss under pressure or the rate of cooking loss. Enhancing meat tenderness and juiciness can be achieved by improving the muscle tethering power. The addition of herbs and bioactive components of herbs to a diet improves the quality of broiler meat by altering the fatty acid content [27]. The findings of this study suggested that the addition of the 0.8% flavored strong powder to the basal diet of the white-feathered broilers inhibited the decline in the muscle pH value and enhanced the a* value, demonstrating a notable trend. The addition of plant-based feed additives to broiler diets considerably enhanced the lean body mass, pectoral muscle tethering force, and leg muscle color a* values while reducing the abdominal fat rate and pectoral muscle meat color L* and b* values. These effects ultimately improved the meat quality of broilers [28]. The research reports that the strong antioxidant and enzyme regulatory activities of Astragalus and Poria enhance antioxidant enzymes and certain metabolic enzymes, effectively inhibiting the generation and transfer of free radicals, reducing the degree of oxidation of muscles, increasing water-holding capacity, and improving the meat color and pH value [29].

The maintenance of host health and immune function largely depends on the intestinal microbiota. However, the precise contribution of specific microbial taxa to the overall function of the intestinal microbiome remains poorly understood. Chickens are commonly used models for studying the intestinal microbiota in poultry, and high-throughput 16S rDNA sequencing has become a valuable tool for assessing its composition and function [30]. The present study demonstrated that the dietary addition of 0.8% of phytogenic feed additive significantly increased the relative abundance and diversity of cecum intestinal flora in white-feathered broilers, as evidenced by α-diversity and β-diversity analyses. The administration of yu ping feng polysaccharide to the basal diet of partridge chickens in Qingyuan not only resulted in an increase in the average daily weight gain of the chickens but also exerted a certain influence on the distribution ratio of the intestinal flora of the chickens [31]. The incorporation of Chinese herbs into the basal diets of broilers enhances the relative abundance and diversity of flora in the jejunum and cecum [32]. Additionally, the research has indicated that compound herbal additives for purple geese effectively stimulate the proliferation of beneficial intestinal bacteria and inhibit the reproduction of harmful bacteria [33]. This study found that at the phylum, genus, and LEfSe levels of analysis of the gut flora, an increase in the relative abundance of *Deinococcus-Thermus*, *Bacteroidetes*, *Actinobacteria*, and *Cyanobacteria* was observed. Bacteroidetes is a beneficial intestinal bacterium. *Bacteroidetes* is involved in the metabolism of carbohydrates and produces beneficial substances, such as short-chain fatty acids, which play a crucial role in maintaining intestinal health [34]. The results of the 16S rDNA functional prediction analysis indicated that white-feathered broilers fed with phytogenic feed additive exhibited significant activation of lipid metabolic pathways in three functional databases, namely COG, KEGG, and Metacyc. This finding suggests that the phytogenic feed additive may promote the activation of the lipid metabolic pathway by modulating the relative abundance of the *Bacteroidetes* phylum in the intestinal contents. Activation of this metabolic pathway provides energy support to the body by regulating the synthesis and utilization of short-chain fatty acids [35]. However, the molecular mechanisms of the activation of lipid metabolism by phytogenic feed additives remain to be further elucidated by experimental verification. *Actinobacteria* is typically not the dominant intestinal microbiota, but its presence is crucial for maintaining the equilibrium and diversity of intestinal microbial communities [36]. It was found that *Deinococcus-Thermus* not only showed significant resistance to environmental extremes but also played an important role in modulating the host immune system [37]. This study further found, through correlation analysis of the previous experimental results, that the correlation between *Deinococcus-Thermus* and immunoglobulin IgM showed a positive correlation, suggesting that *Deinococcus-Thermus* may have a synergistic effect on the immune system of white-feathered broilers and enhance the body’s immune function. Another study has shown that cyanobacteria and their metabolites have significant regulatory effects on the immune function of the organism [38]. Polysaccharides, peptides, and secondary metabolites (phycocyanin) in cyanobacteria can enhance the body’s non-specific and specific immune responses and promote the activity of macrophages, NK cells, and T cells [39,40]. Certain components of cyanobacteria (phycocyanin) have anti-inflammatory and antioxidant effects and can inhibit the release of inflammatory factors (TNF-α and IL-6) and scavenge free radicals, thereby reducing inflammatory responses and decreasing damage to the immune system caused by oxidative stress, thus improving immune function [41]. This study of correlation analysis showed that *Cyanobacteria* was significantly and positively correlated with IgA, an immune indicator of white-feathered broilers, which further confirmed the potential role of cyanobacteria in enhancing the immune function of white-feathered broilers. In summary, the application significance of the results of this study in the field conditions of commercial poultry farms is outstanding, mainly reflected in its convenience, practicality, and affordability, which is in line with the current requirements of food safety strategies.

## 5. Conclusions

This study demonstrated that dietary supplementation with a 0.8% phytogenic feed additive delivered multifunctional benefits in white-feathered broilers, and this operation was simple, convenient, and practical in terms of animal production. Notably, phytogenic feed additive inclusion significantly enhanced the ADG, highlighting its potential as a phytogenic growth promoter. Furthermore, phytogenic feed additive supplementation effectively improved meat quality indices and enhanced the added value by remodeling of gut microbiota ecology and modulating postmortem glycolytic metabolism and lipid peroxidation pathways. Phytogenic feed additive administration induced marked structural shifts in the cecal microbial community, characterized by a substantial increase in beneficial taxa, including *Deinococcus-Thermus*, *Bacteroidetes*, *Actinobacteria*, *and Cyanobacteria*, coupled with suppression of opportunistic pathogens. This prebiotic-like effect may synergistically enhance nutrient absorption efficiency, reinforce intestinal barrier integrity, and stabilize systemic immune homeostasis, thereby mechanistically explaining the observed growth performance improvements. These findings position the phytogenic feed additive as a holistic feed additive in poultry production, aligning with the “multi-component, multi-target” paradigm of traditional Chinese medicine. The synergistic interactions among phytogenic feed-additive-derived phytochemicals likely orchestrated host–microbiota interplay and metabolic reprogramming, offering a sustainable alternative to conventional antibiotic growth promoters. Future investigations integrating metabolomics and transcriptomics are warranted to dissect the molecular mechanisms underlying phytogenic feed additive-mediated microbiota–host crosstalk, particularly its role in regulating key pathways.

## Figures and Tables

**Table 1:** Effects of phytogenic feed additive on the growth performance of white-feathered broilers.

Item	C Group	L Group	M Group	H Group	*p*-Value
Initial weight (g)	51.53 ± 1.10	51.87 ± 0.97	51.92 ± 1.20	52.22 ± 1.29	*p*_1_ > 0.99 *p*_2_ = 0.99 *p*_3_ = 0.93
Final weight (g)	1755.47 ± 54.59	2089.99 ± 56.60 *	2103.06 ± 55.79 *	2108.40 ± 42.74 *	*p*_1_ < 0.001 *p*_2_ < 0.001 *p*_3_ < 0.001
Average daily weight gain (g/d)	40.44 ± 1.60	48.53 ± 1.34 *	48.84 ± 1.35 *	48.96 ± 1.01 *	*p*_1_ = 0.001 *p*_2_ < 0.001 *p*_3_ < 0.001
Average daily feed intake (g/d)	63.94 ± 0.44	66.64 ± 3.65	66.65 ± 5.35	67.05 ± 5.11	*p*_1_ = 0.94 *p*_2_ = 0.94 *p*_3_ = 0.92
Material-to-weight ratio	1.59 ± 0.08	1.37 ± 0.08	1.36 ± 0.07	1.37 ± 0.09	*p*_1_ = 0.20 *p*_2_ = 0.17 *p*_3_ = 0.19

* indicates significant difference with the control group (*p* < 0.05), without * indicates no significant difference with the control group (*p* > 0.05), *p*_1_ represents the comparison between the L group and the C group, *p*_2_ represents the comparison between the M group and the C group, and *p*_3_ represents the comparison between the H group and the C group.

**Table 2:** Effect of phytogenic feed additive on the slaughter performance of white-feathered broilers (%).

Item	C Group	H Group	*p*-Value
Dressing percentage (%)	91.66 ± 0.89	91.76 ± 1.24	>0.99
Semi-clear chamber rate (%)	87.66 ± 0.98	88.21 ± 1.43	0.86
Fully clear chamber rate (%)	75.38 ± 1.16	76.22 ± 1.07	0.70
Pectoral muscle rate (%)	19.82 ± 1.83	20.54 ± 0.61	0.88
Leg muscle rate (%)	29.59 ± 2.80	29.85 ± 3.04	>0.99
Belly fat percentage (%)	1.91 ± 0.22	1.77 ± 0.20	0.83

**Table 3:** Effects of phytogenic feed additive on the meat quality of white-feathered broilers.

Item	C Group	H Group	*p*-Value
1 h pH	6.55 ± 0.11	6.43 ± 0.11 *	0.001
24 h pH	5.96 ± 0.20	5.83 ± 0.14	0.36
1 h L* (the brightness of meat)	53.12 ± 0.85	52.83 ± 1.11	0.89
1 h b* (the yellowness of meat)	23.14 ± 1.2	22.08 ± 0.90	0.10
1 h a* (the redness of meat)	4.86 ± 1.85	6.38 ± 1.28 *	0.11
24 h L* (the brightness of meat)	55.44 ± 1.68	54.22 ± 0.98	0.27
24 h b* (the yellowness of meat)	23.79 ± 1.63	21.59 ± 0.95 *	0.009
24 h a* (the redness of meat)	10.04 ± 3.01	15.75 ± 0.94 *	0.001
1 h Cooking loss rate (%)	15.16 ± 2.53	12.85 ± 3.23	0.56
24 h Cooking loss rate (%)	9.66 ± 1.76	10.52 ± 2.43	0.86
1 h Pressurized water loss rate (%)	20.09 ± 2.33	19.25 ± 2.75	0.96
24 h Pressurized water loss rate (%)	33.55 ± 3.45	33.34 ± 3.58	>0.99

* indicates significant difference with the control group (*p* < 0.05), and without * indicates no significant difference with the control group (*p* > 0.05).

## Data Availability

All data are contained in this article.
